# Relaxation Along a Fictitious Field (RAFF) and Z-spectroscopy using Alternating-Phase Irradiation (ZAPI) in Permanent Focal Cerebral Ischemia in Rat

**DOI:** 10.1371/journal.pone.0069157

**Published:** 2013-07-17

**Authors:** Kimmo T. Jokivarsi, Timo Liimatainen, Risto A. Kauppinen, Olli H. J. Gröhn, Johanna Närväinen

**Affiliations:** 1 Department of Neurobiology, A.I. Virtanen Institute, University of Eastern Finland, Kuopio, Finland; 2 Department of Biotechnology and Molecular Medicine, A.I. Virtanen Institute, University of Eastern Finland, Kuopio, Finland; 3 Clinical Research and Imaging Centre and School of Experimental Psychology, University of Bristol, Bristol, United Kingdom; 4 VTT Technical Research Centre of Finland, Kuopio, Finland; University of Minnesota, United States of America

## Abstract

Cerebral ischemia alters the molecular dynamics and content of water in brain tissue, which is reflected in NMR relaxation, diffusion and magnetization transfer (MT) parameters. In this study, the behavior of two new MRI contrasts, Relaxation Along a Fictitious Field (RAFF) and Z-spectroscopy using Alternating-Phase Irradiation (ZAPI), were quantified together with conventional relaxation parameters (T_1_, T_2_ and T_1ρ_) and MT ratios in acute cerebral ischemia in rat. The right middle cerebral artery was permanently occluded and quantitative MRI data was acquired sequentially for the above parameters for up to 6 hours. The following conclusions were drawn: 1) Time-dependent changes in RAFF and T_1ρ_ relaxation are not coupled to those in MT. 2) RAFF relaxation evolves more like transverse, rather than longitudinal relaxation. 3) MT measured with ZAPI is less sensitive to ischemia than conventional MT. 4) ZAPI data suggest alterations in the T_2_ distribution of macromolecules in acute cerebral ischemia. It was shown that both RAFF and ZAPI provide complementary MRI information from acute ischemic brain tissue. The presented multiparametric MRI data may aid in the assessment of brain tissue status early in ischemic stroke.

## Introduction

Transverse relaxation time (T_2_), diffusion and perfusion MRI are established imaging techniques used in the acute phase of ischemic stroke for both diagnosis and disease prognosis [1]. The challenge in accomplishing both these tasks from a single time point MR session has been addressed by the introduction of multiparametric models for tissue outcome assessment, such as ISODATA [2]. For the determination of tissue outcome from a single MRI session, recent studies using quantitative T_2_ either alone [3] or in combination with T_1ρ_ [4], [5] MRI have shown great promise. Using preclinical models for ischemic stroke, it has been observed that T_1ρ_ contrast shows the lesion within seconds from the onset of ischemia, i.e. the drop in blood flow below the level where the energy state of tissue collapses [6] and the degree of absolute T_1ρ_ change in the early phase of ischemia are in excellent agreement with the extent of neuronal damage which develop in the infarcting brain [7]. A preclinical study addressing the predictive value of multiple MR variables showed that T_1ρ_ and cerebral blood flow (CBF) were the most valuable single parameters in the early hours of ischemia for predicting the long-term tissue outcome [5]. More recently, the increase in T_1ρ_ in the ischemic striatum and cortex of rat has been shown to be linear over the 6 first hour of stroke, revealing the potential to provide information about the duration of ischemia from a single time point MR scan [4]. These observations merit further exploration of rotating frame relaxations for evaluation of acute stroke, despite the fact that the exact mechanisms behind the T_1ρ_ contrast in acute stroke are still much debated [5], [6], [7].

Typically, high specific absorption rate of energy (SAR) is associated with T_1ρ_ MRI measurements, which may limit its clinical use. To reduce SAR and to create such spin-lock contrast with T_1ρ_ and T_2ρ_ contributions, a method entitled Relaxation Along a Fictitious Field (RAFF) was introduced [8]. In an effort to understand biophysical basis of RAFF signal *in vivo*, it has been concluded that the main processes influencing the magnetization in a two-pool system (free water and macromolecule pool) during a RAFF pulse are dipolar and exchange interactions, and the time course of magnetization can be modeled using the Bloch–McConnell equations for longitudinal and transverse magnetization for both of these pools [9]. The exchange processes that may influence the MR signal are the ones occurring at the average RAFF RF amplitude, typically a few hundred Hz and during the refocusing interval, typically a few thousand Hz.

It has been shown that the MT ratio [MTR = (1−M_sat_/M_0_), where M_sat_ and M_0_ are magnetization in the presence and absence of a saturation pulse, respectively] decreases in irreversible ischemia [10], [11] and it shows a different time course from the T_1ρ_ relaxation changes in stroke [12]. It has also been observed that MTR is elevated after a few hours of cerebral ischemia. More detailed Z-spectral data have revealed that the correlation time of the macromolecular motion and the exchange time between the solid and liquid pools of the two-site exchange model have changed approximately an hour after the onset of stroke [12].

**Figure 1:**
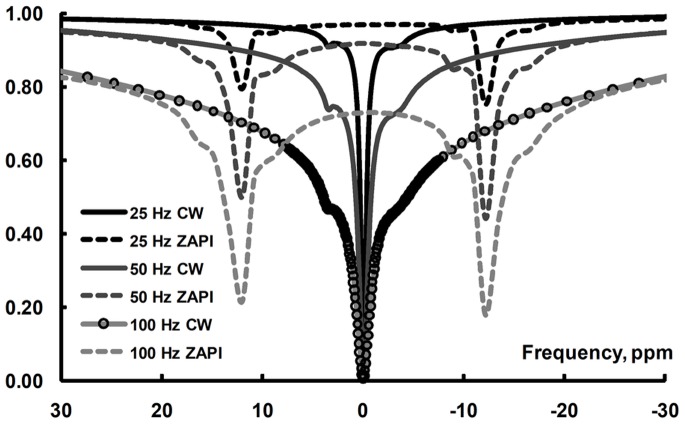
ZAPI, ZAPISM and Z-spectra. To illustrate the ZAPI experiment, ZAPISM and CW Z-spectra, measured at 400 MHz spectrometer (TR 30 s, saturation pulse 15 s, ZAPI-τ 100 µs) from boiled egg white in a 5 mm NMR tube, are shown. The rms saturation amplitude γB_1_ was varied from 25 to 100 Hz. For clarity, the data points are shown as circles for 100 Hz CW curve only. Both CW and ZAPISM follow the same MT Z-spectrum envelope, with direct saturation dips for water (side bands) observed on top of this envelope. Other long-T_2_ spin pools (amides and aliphatic protons) a few ppm from water are observed: on resonance for CW and at the side band frequencies for ZAPI.

An alternative way for detecting MT is Z-spectroscopy using Alternating-Phase Irradiation (with Sine Modulation), ZAPI(SM) [13]. In ZAPI, the irradiation pulse is applied at the frequency of the water resonance and the saturation is targeted to macromolecular spins by using amplitude-modulated RF (alternating phase: AP). With sinusoidal modulation, the frequency profile of a ZAPI pulse displays two off-resonance peaks and the frequency of these depends on the period of the RF modulation. With suitable selection of irradiation power and modulation frequency, direct saturation of water can be tailored to be negligible. Only macromolecular spins will experience saturation and by selection of a range of modulation frequencies, the line shape and T_2_ distribution of the macromolecule pool can be probed. When a ZAPI sequence is run without modulation (i.e. constant wave: CW) at a frequency offset from water, the experiment simplifies into a conventional MT experiment. To illustrate the difference, Figure 1 shows ZAPI and CW Z-spectra measured in boiled egg white with three different RF amplitudes. The details and practical considerations of ZAPI have been discussed in [13].

For this acute cerebral ischemia study we had two aims: first, to investigate the time courses and magnitude of changes in the RAFF and ZAPI parameters, and to compare these to T_1ρ_, T_2_ and MT. Second, to infer the effects of known changes in tissue water content and dynamics to these novel MRI contrasts during acute cerebral ischemia [14].

## Methods

### Ethics Statement

All animal procedures were approved by the Animal Care and Use Committee of the University of Eastern Finland and conducted in accordance with the guidelines set by the European Community Council Directives 86/609/EEC.

### Animal Models

Adult male Wistar rats (280–330 g, n = 9), were subjected to permanent middle cerebral artery occlusion (MCAO) using the procedures described by Longa et al [15]. The occluding thread was left in place for the duration of the MRI scanning and the animal was sacrificed thereafter. Sham-operated animals (n = 2) underwent similar procedure, but without the occlusion. The purpose of the sham animals was only to ensure that the setup is reliable and stable and that the operation produces the expected lesion; the effects of ischemia in the MRI parameters were evaluated by comparing the ipsi- and contralateral values in the experimental animals. All operations and scanning were performed under isoflurane anesthesia with a constant flow of 70/30 N_2_O:O_2_ through a nose cone. The core temperature was monitored online and was maintained close to 37°C by circulating warm water in a heating pad placed under the torso. Breathing rate was also monitored online throughout the MRI study (SA Instruments Inc., NY, USA). Arterial blood gases and pH were analyzed immediately before MR scanning (i-Stat Co., East Windsor, NJ, USA).

### MRI and Data Analysis

The MRI experiments were performed in a horizontal 4.7 T Magnex Scientific Inc. (Yarnton, UK) magnet interfaced to a Varian Unity Inova console (Varian Inc., Palo Alto, CA, USA). MRI was acquired at several time points for of up to 7 hours, with 60 minute intervals during MCAO. A volume coil was used as a transmitter and a quadrature half-volume coil as a receiver (Rapid Biomedical GmbH, Rimpar, Germany). Except for diffusion, a fast spin-echo (FSE) readout (128×64, echo spacing 10 ms, FOV 25.6×25.6 mm^2^) was used for all imaging with different preparation blocks. In all cases the data was collected from a single axial slice positioned in the middle of the striatum, 5 mm caudally from the olfactory bulb.

The trace of the diffusion tensor (D_av_ = (1/3) Trace(D)) image was used to localize the acutely ischemic tissue. D_av_ was quantified as an average of diffusion values measured along the three orthogonal directions (b-value 356 s/mm^2^) using a spin-echo sequence (time to repetition (TR) 1 s, time to echo (TE) 35 ms). Cerebral blood flow maps were acquired with a continuous arterial spin labeling technique with a 3 s labeling pulse (γB_1_ = 200 Hz) applied 2 cm from the imaging slice, and a 500 ms post-labeling delay [5]. Eight label-control pairs were used to estimate CBF using a water partition coefficient, λ, of 0.9.

T_1ρ_ MRI maps were collected with an on-resonance continuous wave (CW) spin lock (SL) preparation block AHP-SL-AHP (AHP = adiabatic half passage). The duration of the SL pulse ranged from 8 to 64 ms with SL amplitude (B_1,SL_) of 1700 Hz (40 µT), repetition time (TR) of 2.5 s. T_2_ was measured with an adiabatic double refocusing block, with an echo time (TE) of 8–64 ms, TR 2.5 s. T_1_ was measured using an inversion recovery sequence with five inversion times (TI 5–1500 ms, TR 3 s). All relaxation maps were calculated using three-parameter nonlinear fits.

For measuring T_RAFF_, the sine/cosine pulses with the previously described pulse design [8] were used, with a peak amplitude (γB_1_) of the RAFF pulse train of 625 Hz and five RAFF pulse train durations, linearly spaced between 0 and 144 ms. Two measurement, with and without initial inversion (HS1-pulse, T_p_  = 4 ms, γB_1_ = 2.5 kHz) were performed and simultaneous fitting to the non-zero steady state model [8] was applied to the data. Fitting of T_RAFF_ was performed on pixel-by-pixel basis to obtain T_RAFF_ maps. The Bloch–McConnell equations for signal decay during the RAFF pulse train were used to simulate T_RAFF_ change at two time points during stroke [8]. In these simulations, the values for relaxation times, pool sizes and exchange dynamics were incorporated from earlier work [16], in which a two-site exchange (2SX) model was fitted to the values of T_1ρ_ and T_2ρ_ in the striatum in the identical middle cerebral artery occlusion model. As the simulation model is a simplification of conditions prevailing *in vivo*, two further simulations were run to test how the results change in more realistic simulation conditions. To mimic diffusion of water in field gradients, e.g. around capillaries, the water frequency was allowed to vary sinusoidally during the RAFF pulse, with an oscillation amplitude of 40 Hz and an oscillation frequency of 1, 10, 100 or 1000 Hz. To model the effect of finite water line width, the simulations were run for 101 isochromats representing a 40 Hz FWHM Lorentzian line shape. Each isochromat was computed separately and the sum over the isochromats was used to calculate T_RAFF_. In both of these simulations, the 2SX parameters were the values reported in [16] for the contralateral tissue. As such, the results are reported as differences from the values given by the 2SX simulation using infinite narrow lines and without B_0_ oscillation.

For ZAPI, a saturation pulse with duration of 7 s was used. For the CW experiment, a train consisting of block pulses of constant phase and without delays between pulses was used, resulting in a conventional MT experiment. CW was measured with saturation pulse offsets of 50 kHz (a reference to which all ZAPI data were normalized), −5 kHz (−25 ppm) and −10 kHz (−50 ppm). For the AP (alternating phase) experiment, the amplitude of the irradiation was sinusoidally modulated so that the phase was inverted for each lobe. For AP, the saturation was applied on the water resonance. To control the T_2_ filter, the subpulse length τ was varied from 100 µs, a value shown earlier to result in the same amount of MT as the CW experiment with the same root mean square power [13], down to 80, 60 and 40 µs. For CW, the saturation amplitude was 50 Hz. AP was run with the same root mean square power, which is 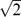 higher in amplitude to compensate for the amplitude modulation. Image intensities from the ROIs were first normalized to the intensity of the same ROI in the reference scan at 50 kHz offset and MTR values were calculated. To estimate the effect of the T_2_ filter using RF modulation frequency the ratio of normalized ROI signal intensities S(n µs)/S(100 µs) was used.

A B_1_-map was calculated for the homogeneity control of the RF field. A cosine function was fitted to signal intensity oscillation that was caused by a variable length square preparation pulse and a crusher gradient in front of a FLASH pulse sequence (TR 4.5 ms, TE 2.2 ms), as previously described in [5].

**Figure 2:**
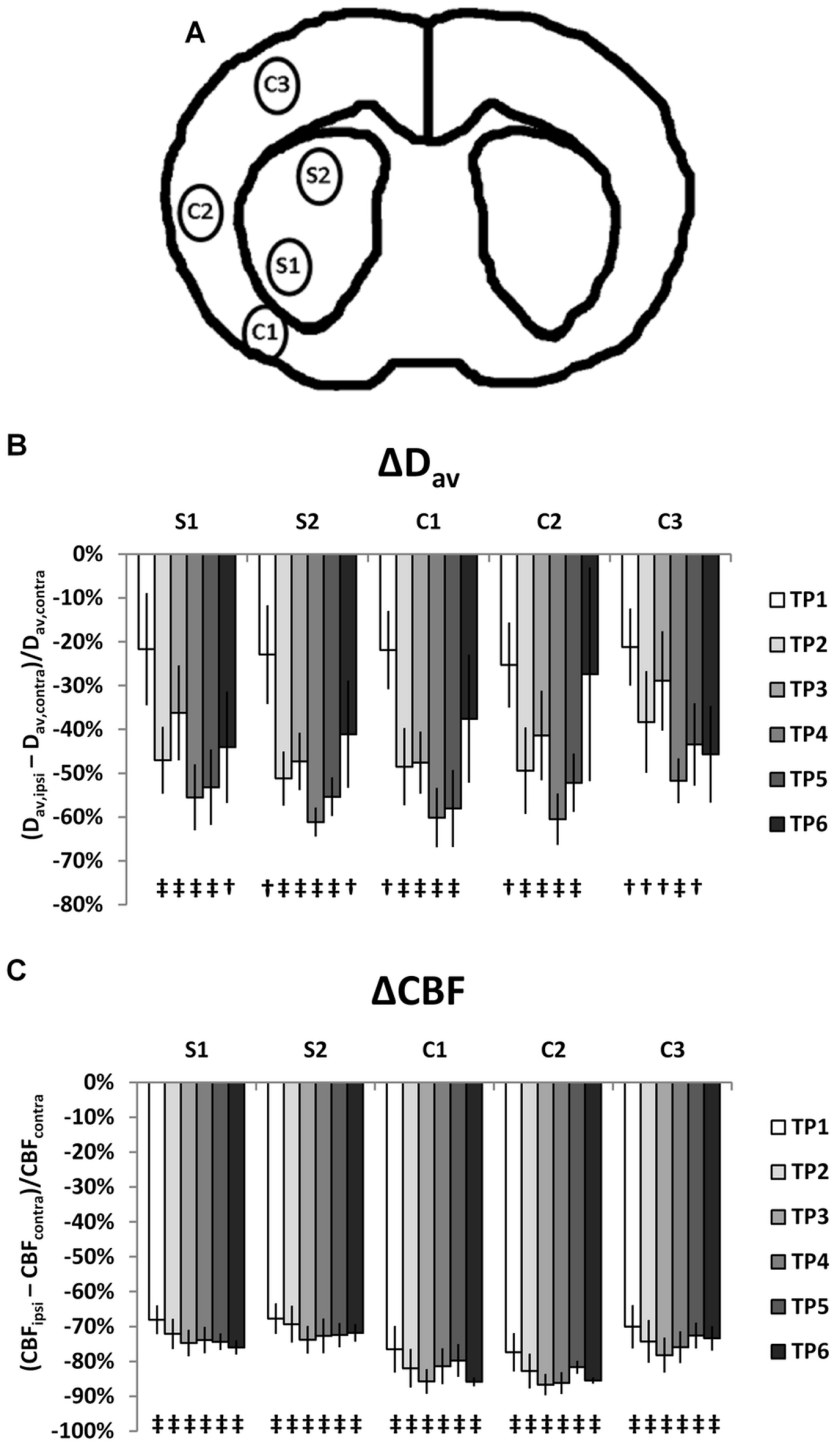
Brain regions, blood flow and apparent diffusion changes. The five brain regions used in the MRI data analysis (A), the time courses of change in CBF measured by arterial spin labeling (B), and apparent diffusion change, D_av_, (C) in three of these areas: in stroke (solid markers) and in sham-operated animals (open markers). All changes are relative: (ipsi-contra)/contra.

Values for each MRI variable were calculated as the mean of small ROIs (approximately 3 mm^2^ in-plane) positioned in the ischemic lesions in the striatum (S1 and S2) and basal cortex (C1, C2, and C3, Fig. 2A). There were some variations in the stroke lesion sizes. The lesion covered the whole striatum in 7 cases and in 5 cases the cortex from C1 to C3. The locations for the ROIs were chosen visually to evaluate the growing ischemic lesion and thus the different phases of infarct progression. It has been shown that in the striatum and cortex, ischemia evolves with differing time courses for T_1ρ_ and T_2_ [17]. Corresponding contralateral ROIs were chosen as reference. The change in MRI parameters were then calculated as the difference between the mean ipsilateral and contralateral values and normalized to the contralateral values. To characterize and compare the dynamics of T_RAFF_ during the ischemia, correlation coefficients between the changes, pooled from all brain regions, in T_RAFF_ and in the other relaxation parameters were computed. The signal-to-noise of the parameter maps was estimated from a ROI located on a homogeneous brain region (striatum) by sqrt(2)*S_ROI_/σ_noise_, where S_ROI_ the average signal in an ROI and σ_noise_ the standard deviation of the signal in a homogenous region. All calculations were made using Aedes software (http://aedes.uku.fi) in Matlab platform (MathWorks, Natick, MA, USA). All values are shown as mean ± standard error of mean (SEM).

## Results

In the sham-operated animals, no systematic ipsi-contra differences in any MRI parameters quantified were seen. The values for MT parameters and relaxation in sham animals were similar to the values in the contralateral side in the ischemia group. In stroke animals, the average D_av_ decreased by 42±9% (from the average value of 0.94±0.11×10^−9^ m^2^/s in the contralateral hemisphere) by 100 min of MCAO, indicating severe ischemia in all brain regions studied. The apparent diffusion in normal brain was slightly higher than the generally reported which could have been due to the relatively low b-value range used. However, the diffusion drop caused by ischemia was typical for severe ischemia. Diffusion remained low throughout the observation time but some recovery (up to –10% of the contralateral value) in the peripheral brain region C3 was observed. CBF values were reduced in all ROIs (mean over all ROIs was 76±5% lower than the average value of 230±40 ml/100 g/min on the contralateral side) at the 100 min time point, showing excellent reproducibility of the MCA occlusion. CBF remained at this low level in all brain regions studied. D_av_ and CBF time courses are presented in Fig. 2 B–C. During the experiments, the core temperature of the animals was 37.0±0.3°C and the breathing rate was 60±4 min^−1^. The pO_2_ and pCO_2_ of venous blood before the MRI were 100±7 mmHg and 61±3 mmHg, respectively.

**Figure 3:**
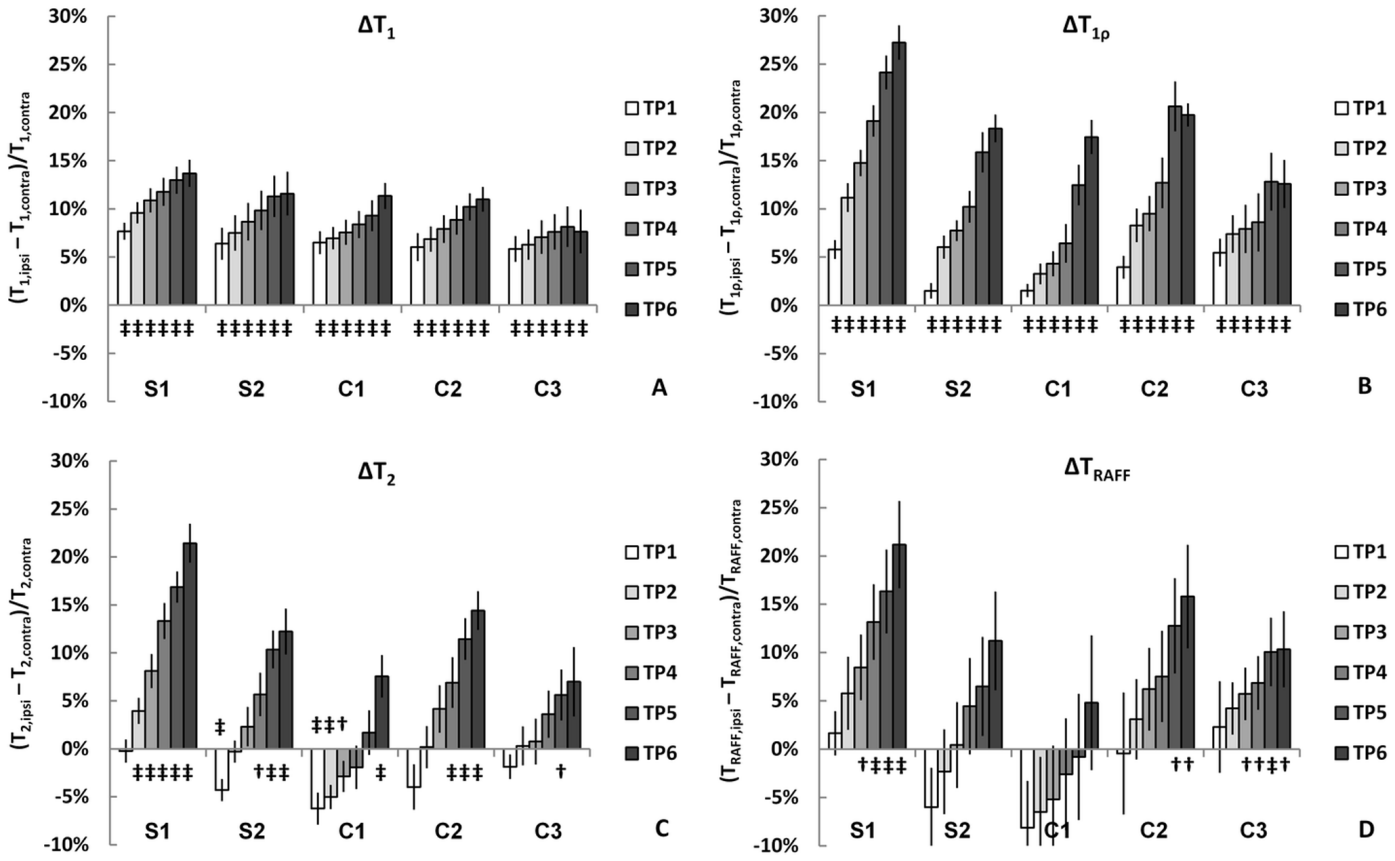
The temporal evolution of the relative differences in relaxation rates. Data are shown from time point one (TP1) to six (TP6) between the hemispheres in the five brain regions (S1-C3) studied. The results from Student’s t-tests are given below the graphs: p<0.05 =  † and p<0.01 =  ‡.

**Figure 4:**
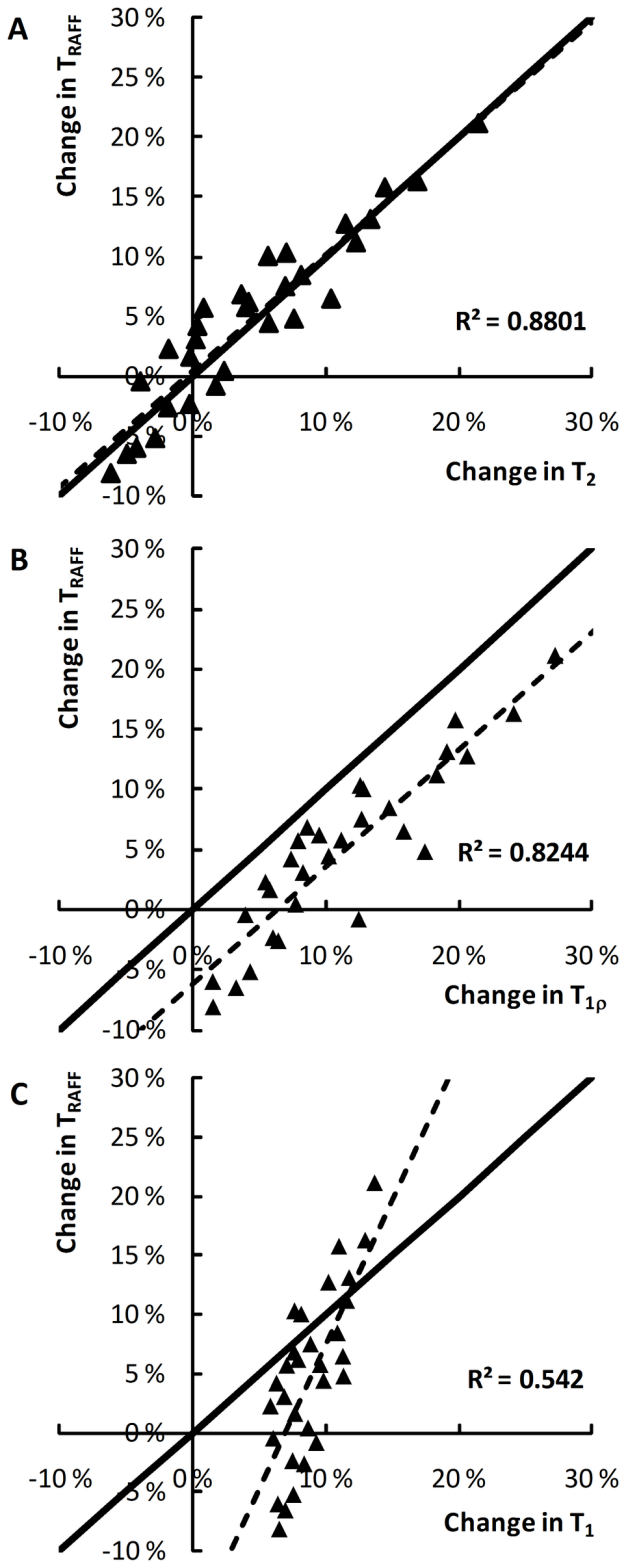
Correlation between changes detected by RAFF and other relaxation times. The data are pooled from all time points from the five brain regions studied. The regression lines (dashed, R^2^ values shown) show high correlations for all parameter pairs; however, the pair T_2_-RAFF is the best match to the line of unity (solid line), confirming the similarity of the time courses.

The baseline relaxation times measured in sham-operated animals are given in Table 1. As expected, T_1_ and T_1ρ_ were already elevated in the ischemic lesion at the first time point and increased linearly as a function of time (Fig. 3A and B). Similar progressive lengthening was observed in T_2_, but the onset of increase occurred approximately an hour after induction of ischemia (Fig. 3C). The delay, or even an initial shortening of T_2_, has been associated with increased capillary and venous deoxygenation and blood volume, resulting from compromised but non-zero blood flow in and near the ischemic region [17]. T_RAFF_ displayed a time course more resembling that of T_2_ than of T_1_ (Fig. 3D). The sensitivity of different relaxation times to ischemia was quantified by analyzing the respective correlations. When the brain areas were analyzed separately, for all parameter pairs the R^2^ values were high (0.87 or higher), as all parameter changes were close to linear. When the samples were pooled (data from the five brain areas combined, Fig. 2A), the correlation between T_1_ and RAFF was steeper than that of RAFF and T_1ρ_ and RAFF and T_2_, due to the smaller range in T_1_. Furthermore, the regression lines (Fig. 4) show that the correspondence between RAFF and T_2_ is very close to the line of unity, whilst other relaxation parameters either correlate with offsets or with different slopes.

**Table 1:** The measured relaxation rates in the sham-operated animals.

Brain region	T_1_ [ms]	T_2_ [ms]	T_1ρ_ [ms]	T_RAFF_ [ms]	MTR CW 10 kHz [%]	MTR CW 5 kHz [%]	MTR ZAPI 100 µs [%]
**S1**	1070±18	57.0±1.0	73.6±1.4	114.9±2.3	15.6±1.1	28.6±1.3	16.9±1.7
**S2**	1086±18	56.8±1.5	73.8±1.8	118.9±4.5	15.4±1.4	27.9±1.3	16.5±1.9
**C1**	1139±29	64.4±2.1	83.8±2.2	133.7±6.5	14.7±1.7	26.0±1.8	15.2±2.4
**C2**	1120±15	55.7±0.9	72.7±2.2	112.1±3.7	15.9±1.9	27.9±1.6	16.3±2.5
**C3**	1092±19	54.8±0.8	71.6±1.5	107.8±2.8	15.8±1.9	28.3±1.4	16.9±1.8

The figures presented are mean values of areas in both hemispheres and the six time points. No systematic variation across the hemispheres, between the time points or between the animals was observed. These values agree closely with the relaxation data measured in the contralateral hemispheres of the stroke animals as well.

RAFF simulations were performed to predict the change in T_RAFF_ in two time ranges: (a) from pre-ischemic state at 0 min (estimated by contralateral values) to 135 min, and (b) from 135 min to 245 min. Due to limitations of the model in taking into account variety of relaxation mechanisms in tissue, the baseline of T_RAFF_ is inexact, predicting a value of 102 ms for pre-ischemic condition, while the measured value is 120 ms. However, the computed changes in T_RAFF_ in the ischemic tissue (6.5% for (a) and 6.6% for (b)) are in excellent agreement with the values observed in the S1 region (Fig. 3D). When the simulations were run with oscillating water frequency, mimicking diffusion in field gradients around capillaries, the computed T_RAFF_ was further decreased (94, 98, 100 and 100 ms, for oscillations frequencies of 1, 10, 100 and 1000 Hz, respectively). The simulation for the system of finite line width also decreased the value of computed T_RAFF_ giving a value of 100 ms.

**Figure 5:**
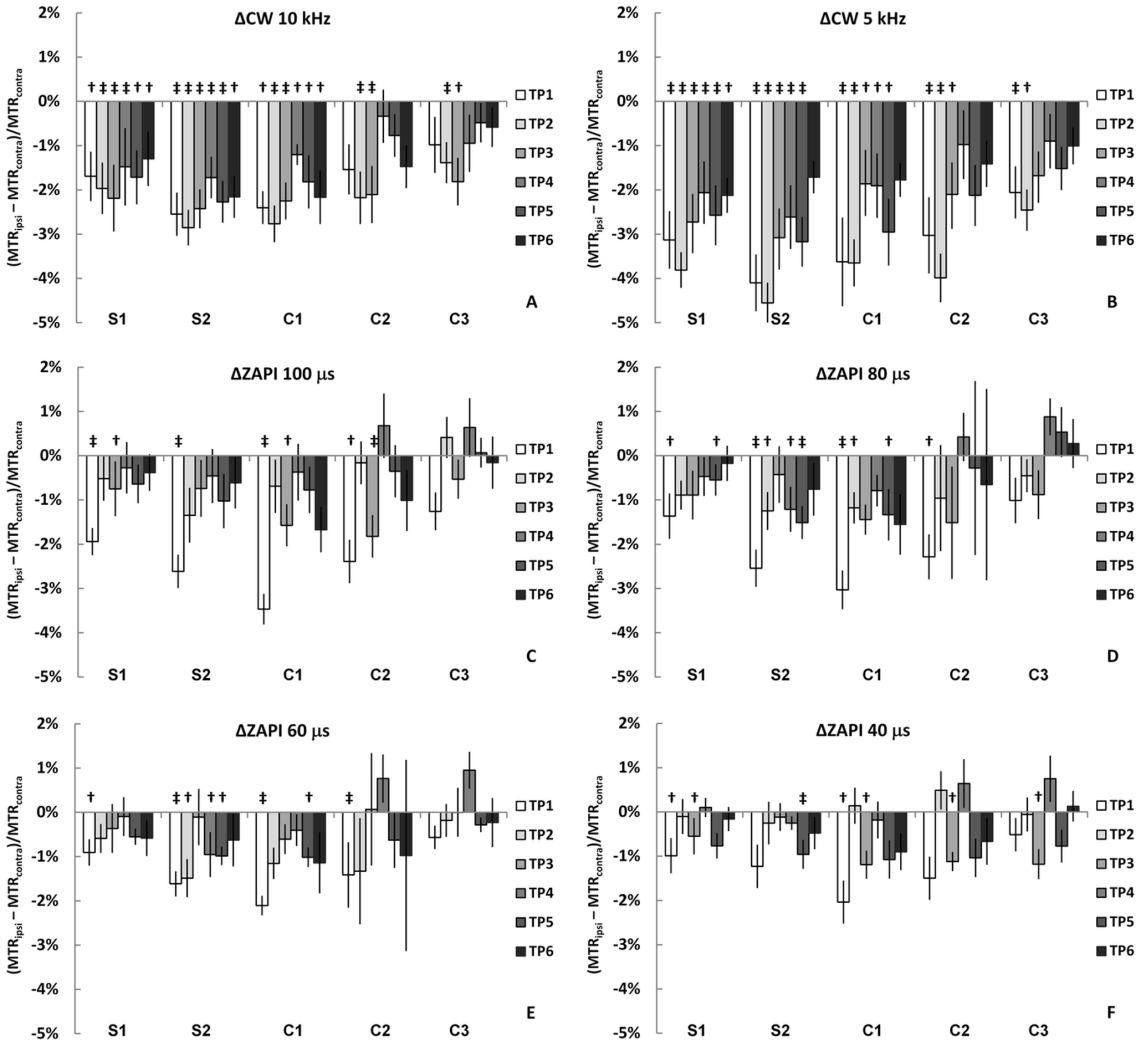
Progression of the MT changes for conventional MT and ZAPISM. Conventional MT (marked CW) is shown at offsets of 10 and 5 kHz, and ZAPISM (marked AP) on resonance, with full MT (τ  = 100 µs) and with T_2_ filtering (τ  = 80, 60 and 40 µs). The values are (ipsi-contra)/contra changes at brain regions S1-C3, at time points one (TP1) to six (TP6). The results from Student’s t-tests are given by: p<0.05 =  † and p<0.01 =  ‡.

During ischemia, MT signals, as detected by ZAPI and CW-MT, were increased at the first time point. The time courses for MT parameters are presented in Figure 5. CW-MT (Fig. 5A and B) shows larger differences between the hemispheres than ZAPI MT parameter (Fig. 5C), most likely due to early negative BOLD effect (resulting in line broadening) and changes in T_1_ relaxation contributions to MT via direct saturation of water. However, there was no further increase in ZAPI MT parameter during the evolution of the stroke. This is strikingly different to the time course of all the relaxation parameters, especially T_1ρ_. This suggests that neither RAFF nor T_1ρ_ are MT-driven relaxation processes. The uncoupling was confirmed by correlation analysis in which changes in MT parameters and relaxation times were compared. All R^2^ values, ranging from 0.10 to 0.39, for linear correlation were positive but statistically insignificant.

**Figure 6:**
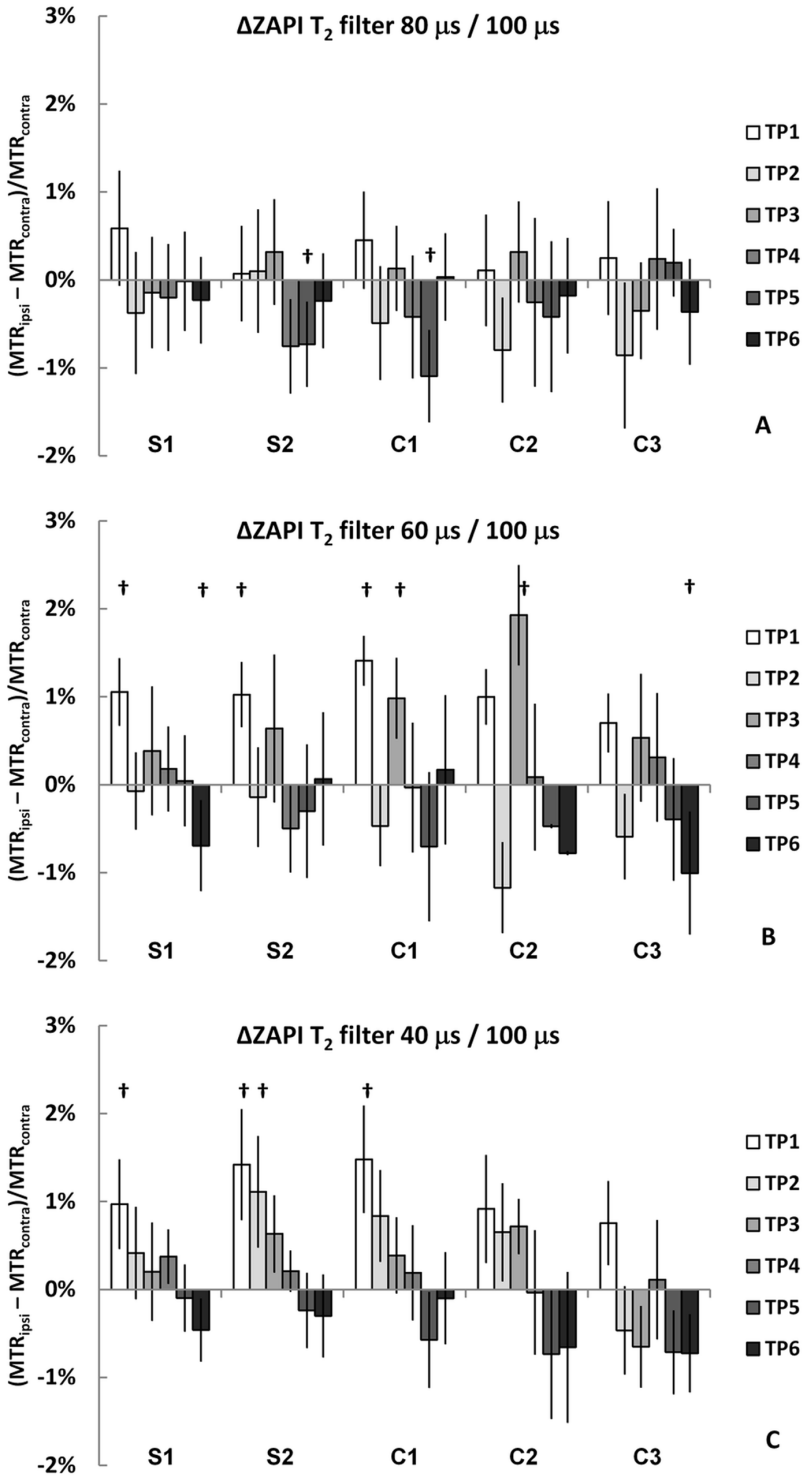
Time courses for relative ipsi-contra differences in T_2_-filtered ZAPISM MT. The values of parameter S(n µs)/S(100 µs) are shown at time points one (TP1) to six (TP6) in the different brain regions S1-C3. The results from Student’s t-test are given by: p<0.05 =  † and p<0.01 =  ‡.

Removing the longer macromolecular T_2_ components from the MT process with the T_2_ filter in ZAPI (Fig. 5D–F), showed a similar course to the conventional MT: The ZAPI MT parameter decreases at the first time point and remains low throughout all time points studied. The amplitude of change is naturally smaller due to the fact that the overall MT is smaller with stricter filter (macromolecules with shorter T_2_), because fewer macromolecular protons contribute to the signal. A simple measure of macromolecular T_2_ distribution is S(n µs)/S(100 µs), in which n  = 80, 60 or 40 µs. In ischemic tissue (Fig. 2A: S1, S2, C1) this parameter for τ of 60 and 40 µs was significantly increased at the 60 minute time point (Fig. 6 B and C), which may suggest early changes in macromolecular dynamics in ischemic brain parenchyma. The direction of change indicates that while the ZAPI MTR is increased, i.e. S(100 µs) is decreased, there is no corresponding decrease in signal measured with the selection of macromolecules with shorter T_2_. These differences were reversed as the stroke evolved, and the few significant data points in later stages of ischemia actually represent cases where S(n µs)/S(100 µs) decreases. This trend is clearly visible in the data for τ  = 40 µs (Fig. 6 C), even if all the points are not statistically significant.

**Figure 7:**
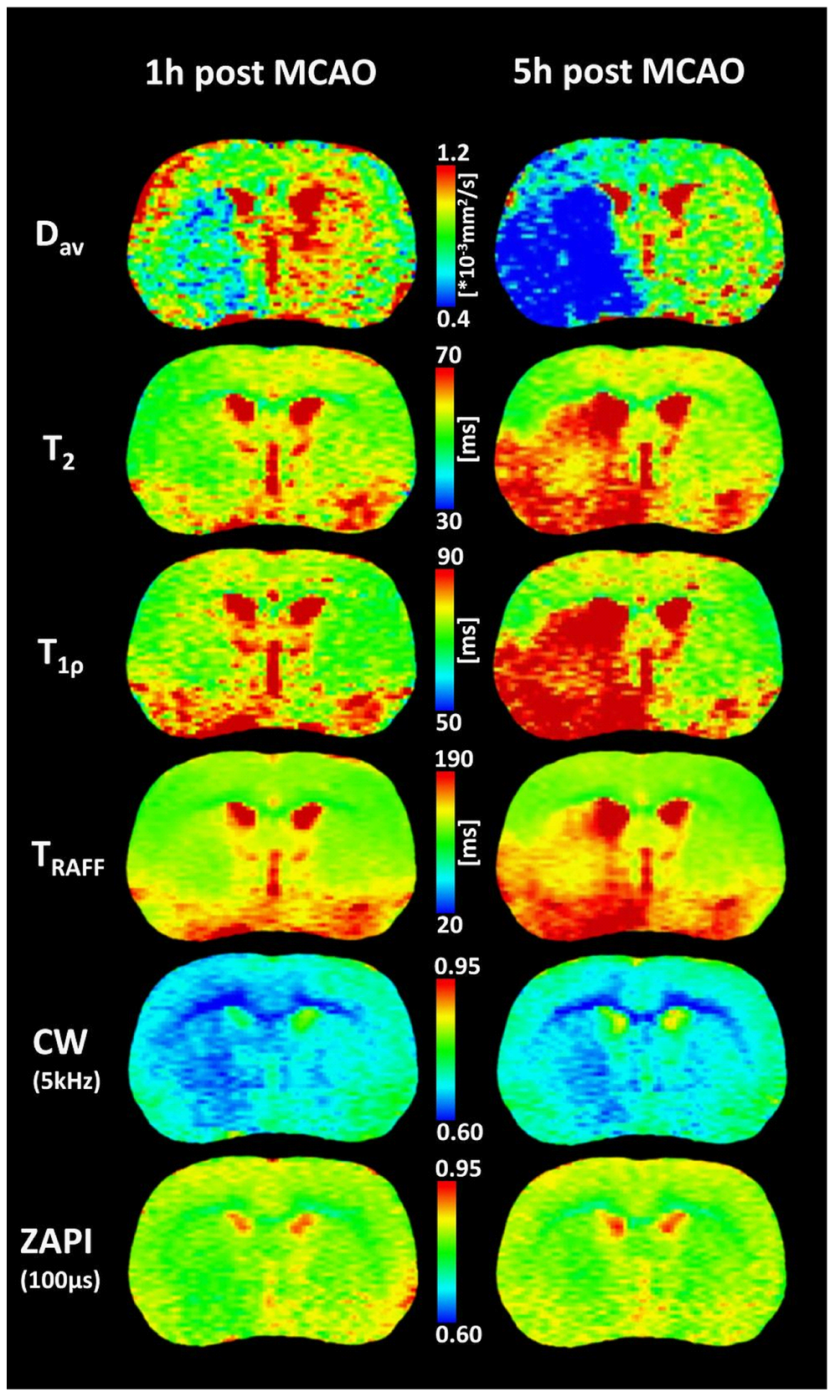
Representative diffusion (D_av_), T_2_, T_1ρ_ and T_RAFF_ maps and CW-MT and ZAPI MR images. Data from one animal acquired at 1 and 5 hours post MCAO. Ischemic lesion is visible in the left hemisphere.

Figure 7 shows sample images of one animal imaged at one and five hours post MCAO. Signal-to-noise ratios for the MRI parameter maps were as follows: T_1_ 58, T_2_ 56, T_1ρ_ 43, T_RAFF_ 44, D_av_ 10 and CBF 15. For CW-MT and ZAPI the corresponding ratios for MT-maps varied between 86 and 135.

## Discussion

We have characterized the time courses for RAFF and T_1ρ_ relaxation parameters, as well as ZAPI and conventional MT, in the acute phase of permanent focal cerebral ischemia in rat. It is evident that the temporal evolutions of the relaxation parameters studied are unrelated to MT-driven processes as detected by CW-MT MRI. We show that, T_RAFF_ in brain parenchyma can be described by Bloch–McConnell equations and the changes caused by ischemia in T_RAFF_ are predicted accurately by these equations using a two pool model [8]. The current data demonstrate that the ZAPI MT parameter is elevated in early minutes of ischemic stroke, and furthermore, the data measured with the ZAPI T_2_ filter suggest changes in macromolecular T_2_ distribution during the evolution of stroke. Overall, the MRI data obtained may provide complementary information for tissue status assessment for established MRI techniques used to image acute ischemic stroke.

In ischemic stroke, the supply of oxygen is severely compromised or completely interrupted, leading primarily to a collapse of the energy state followed by a cascade of damaging events in the brain tissue. Several simultaneous processes occur with different timescales, such as membrane depolarization, ionic and water shifts between extracellular and intracellular space, and activation of destructive proteases and lipases. A steady increase in longitudinal relaxation throughout the observation time window is evident, chiefly due to increased overall water content. Unlike T_1ρ_ and T_2_, T_1_ is already elevated at the first time point and the slope of subsequent increase is smaller than that of T_1ρ_ and T_2_. A similar linear increase is seen in transverse relaxation, but in some brain regions this is preceded by an early reduction (Fig. 3C). The diffusion recovery in the peripheral C3 region in the last time point (Fig. 2B) is reflected in longitudinal relaxation parameters (Fig. 3A and B).

The temporal evolution of T_RAFF_ in stroke resembles that of transverse relaxation (Fig. 3D). Indeed, T_RAFF_ relaxation is accelerated during the first hour of ischemia, especially in S2 and C1 regions, (Fig. 3D). The initial decrease and/or plateau in T_2_ [17] is associated with high concentration of deoxygenated hemoglobin and dilated microvessels in the poorly perfused, but still viable, tissue in the early minutes of the insult. In such a matrix, the apparent T_2_ measured from the parenchyma is a net result of shortened blood T_2_, increased cerebral blood volume (CBV) and accelerated dephasing by extravascular field gradients. In this application of RAFF, half of the magnetization relaxes via T_2ρ_ type relaxation and it seems that the RAFF contrast in stroke is dominated by this relaxation pathway. However, when the T_2ρ_ is measured using a train of adiabatic pulses, the plateau evident in T_2_ disappears [16]. The difference between RAFF and T_2_ MRI may be due to selection of B_1._ In our study the value of γB_1_ was only 20% of the value used previously in [16].

The measured values of T_RAFF_ are longer than the simulated relaxation times. Our RAFF simulations employ a classical two-site exchange model with six (2×3) differential equations. The model assumes that the chemical shift between water and macromolecules remains constant and that both pools have fixed Larmor frequencies. However, when the simulations are run for a more realistic model system, the number of relaxation channels is increased and the net effect is that T_RAFF_ is further decreased. Adding complexity into the model will not explain the difference between observed and simulated RAFF baseline, but its cause must lie in the relaxation model itself and/or values for relaxation parameters used in the simulations. However, despite this offset in absolute values of T_RAFF_, the changes in T_RAFF_ in stroke are predicted by the simulations.

Conventional MT reflects a variety of processes related to macromolecule–water interaction, mainly via dipolar through-space effects, direct saturation and also the dynamics and structure of the macromolecules. In early stroke, MTR in brain tissue increases, i.e. the saturation pulse delivered at an offset frequency causes more signal loss than in healthy brain [10]. However, measuring pure MT is challenging, as the direct saturation of the water line interferes with the signal, especially when high RF irradiation amplitudes are used. This is the probable reason both for increased sensitivity of −5 kHz irradiation offset MT compared to −10 kHz and for ZAPI; changes in water relaxation rates are reflected in the water line width, and consequently in the amount of direct saturation. The effect of relaxation rate changes is also the probable cause behind the time-dependent trend observed in the −5 kHz MT data. Another issue in measuring MT is the contribution from magnetization exchange between water and other long T_2_ spin pools, such as amides and CH-proton signals. These contributions in a Z-spectrum are, however, offset frequency dependent and are only observed a few ppm from water.

The ZAPI experiment can be considered as a dual off-resonance irradiation [13]. Side lobes fall at ±1/(2 τ), which is ±5 kHz for τ of 100 µs, and with decreased τ, the off-resonance frequency increases. Side lobes experience lower B_1_ than the nominal irradiation B_1_, and thus the direct saturation lines are narrower and contamination from one side lobe is less than what is observed in conventional MT at the corresponding off-resonance frequency. Even if there are two side lobes in ZAPI, the ZAPI experiment results in higher signal than a conventional off-resonance MT experiment with similar rms power, Fig. 1 and [13], suggest that the direct saturation component is smaller in ZAPI. The dual direct saturation contribution in ZAPI is an advantage in the case of B_0_ shifts across the sample: in conventional MT the amount of direct saturation will change as a function of water resonance, while in ZAPI the changes in the direct saturation from the two side lobes will act to balance each other, as long as the B_0_ inhomogeneity remains small compared to the offsets of the irradiation. These practical aspects of ZAPI experiments have been discussed in more detail in [13].

The time courses for ZAPI and conventional MT differ in acute ischemia. This may be due to the influence of relaxation changes which influence the standard off-resonance CW-MT signal more than the ZAPI signal. According to ZAPI T_2_ filter experiments, there is a shift in the T_2_ distribution of the macromolecular pool at the early time points of acute stroke: the ratio between signal with ‘T_2_-filtered’ MT and full MT increases (Fig. 6). This may be a result of an increase in ZAPI MTR, as S(100 µs) decreases whilst S(<100 µs) remains unchanged. However, previous MT work [12] revealed apparent T_2_ changes in the liquid pool only, but no change in the macromolecule pool. This suggests that the changes in ZAPI originate from modification in the distribution of shorter T_2_ components of the macromolecules. This change is not observed throughout the evolution of the stroke, in fact there is a shift to the opposite direction in the last two ZAPI MTR time points. The changes detected by the ZAPI T_2_ filter are small, much smaller than the inherent differences in T_2_ seen between brain and muscle tissue [13]. However, the ZAPI technique is sensitive to these small changes and may prove a useful detection tool in other conditions in which the changes in macromolecular composition and consequently in T_2_ distribution and line shape are larger.

An important technical note is that both MT and RAFF MRI are more demanding for B_1_ homogeneity than the other relaxation parameters used in this study. Potentially, B_1_ inhomogeneity induces additional noise when averages from different experiments are computed. However, there was no systematic difference between the hemispheres in the sham-operated animals and therefore, B_1_ homogeneity must have been high. Another issue, which is related to demanding RF pulse sequences, is tissue heating. In both RAFF and ZAPI the root mean square RF amplitudes are very small, however, which keeps the SAR low.

The entire MRI protocol took about 50 minutes to acquire. The protocol in this study was not optimized for temporal resolution, but rather we wanted to run the MT experiments at steady state. The images were acquired with a segmented FSE readout. For all these methods used, there are faster acquisition paradigms and pulsed MT techniques that can be applied if time is paramount. The slow acquisition also means that the time points for different parameters do no match exactly; the relaxation times were measured first, followed by CW-MT and ZAPI-MT. However, as the order was kept the same and six time points were measured, we believe this is not a serious problem for interpretation and comparison of the time courses.

In conclusion, we have shown that both RAFF and ZAPI provide complementary MRI information from the acutely ischemic rat brain tissue. From a mechanistic point of view, MT and relaxation changes during evolving stroke are not correlated. Early ZAPI signal changes reflect altered macromolecular T_2_ distribution rather than net water accumulation. The multiparametric MRI results may be useful for multiparametric assessment of brain tissue status early in acute ischemic stroke.
